# Synthetic Strategies to Terpene Quinones/Hydroquinones

**DOI:** 10.3390/md10020358

**Published:** 2012-02-14

**Authors:** Marina Gordaliza

**Affiliations:** Farmacy Faculty and Institute of Science and Technology Studies, Campus Miguel de Unamuno, Salamanca University, 37007 Salamanca, Spain; Email: mliza@usal.es; Tel.: +34-923-294528; Fax: +34-923-294515

**Keywords:** terpene quinone, terpene hydroquinone, synthesis, chemical modification

## Abstract

The cytotoxic and antiproliferative properties of many natural sesquiterpene-quinones and -hydroquinones from sponges offer promising opportunities for the development of new drugs. A review dealing with different strategies for obtaining bioactive terpenyl quinones/hydroquinones is presented. The different synthetic approches for the preparation of the most relevant quinones/hydroquinones are described.

## 1. Introduction

The chemical substances from plants and animals have been and remain to be an important source of drugs and products used in food products, cosmetics and agriculture, amongst other fields. Natural compounds offer an enormous structural diversity and in some cases, a big biological power and thus it is unlikely that the chemistry of synthesis can replace cellular biochemistry as the source of new compound. These observations, in addition to the enormous biodiversity of the planet (plants, sea and microorganisms), which is in a lot of cases inexplored, point to natural compounds as a promising source of drugs [1,2,3,4,5,6,7,8,9,10,11,12,13,14,15,16,17,18,19,20,21,22,23,24,25,26,27,28,29,30,31,32,33,34,35,36,37,38,39,40,41,42,43,44,45].

Natural products have been a rich source of agents of value to medicine. More than half of the currently available drugs are natural compounds or are related to them, and in the case of cancer this proportion surpasses 60% [4,8]. This situation is accompanied by increasing interest from drug companies and institutions devoted to the search for new drugs [46,47]. Additionally, many new natural compounds of diverse structures have been considered *prototypes*, *leads* or *heads of series* and their later structural modification has afforded compounds with pharmacological activity and extraordinary therapeutic possibilities [11,22,25,26,48].

The research in natural compounds, which is continually expanding and is of enormous interest, explores new compounds coming from different sources, among which the sea could be considered as an almost infinite source of natural resources, some of which have important therapeutic potential [49,50,51,52,53,54,55,56,57,58,59,60,61,62,63,64,65,66,67,68,69,70,71,72,73,74,75,76,77,78,79,80,81,82,83,84,85,86,87,88,89,90,91,92,93,94,95,96,97,98,99,100,101,102,103,104,105,106,107,108,109,110,111,112,113,114,115,116,117,118,119,120,121,122,123,124,125,126]. The discovery of drugs from marine natural products has enjoyed a renaissance in the past few years. Ziconotide (Prialt^®^; Elan Pharmaceuticals), a peptide originally discovered in a tropicalcone snail, was the first marine-derived compound to be approved in the United States in December 2004 for the treatment of pain [127]. Combination of ziconotide and morphine allows safe and rapid control of oral opioid-refractary malignant pain [128]. In October 2007, trabectedin (Yondelis^®^; PharmaMar) became the first marine anticancer drug to be approved in the European Union. Trabectedine is an intravenous antineoplastic agent originally derived from tne Caribbean marine tunicate *Ecteinascidia turbinata* and now produced synthetically [129]. Trabectedine shows variable levels of activity against several types of solid tumor including soft tissue sarcoma, ovarian cancer, breast, melanoma, non small lung cancer, prostate and endometrial cancer [130,131,132]. The drug is especially active in leiomyosarcoma and liposarcoma and is a therapeutic option in the palliative approach to the metastatic uterine leiomyosarcoma patient [133]. Eribulin mesylate (E7389), designed by the Japanese laboratory Eisai (Eisai Research Institute, Andover, MA, USA), shows antitumor properties for the treatment of breast cancer [134]. This is a synthetic analogue of the natural product halichondrin B, isolated from *Halichondria okadai (Lissodendoryx* sp.), a marine sponge commonly found in Japanese seas; its antitumor activity was discovered in 1986. Eribulin binds to the vinca domain of tubulin and inhibits the polymerization of tubulin and theassembly of microtubules, resulting in the inhibition of mitotic spindle assembly, the induction of cell cycle arrest at G2/M, and, potentially, tumor regression. Eribulin mesylate is now in phase II clinical trials and is active in metastatic or locally advanced breast cancer [135,136,137,138].

Excellent reviews on natural compounds of marine origin have been published [49,50,51,52,53,54,55,56,57,58,59,60,61,62,63,64,65,66,67,68,69,70,71,72,73,74,75,76,77,78,79,80,81,82,83,84,85,86,87,88,89,90,91,92,93,94,95,96,97,98,99,100,101,102,103,104,105,106,107,108,109,110,111,112,113,114,115,116,117,118,119,120,121,122,123,124,125,126] that explore the taxonomy, structural elucidation, role of databases, biosynthetic studies, biomedical potential, synthesis and the technologies necessary for advancing bioactive marine natural product lead compounds into actual pharmaceuticals. Amongst these, the recently published review by Fattorusso *et al.* [124] particularly stands out.

Among the natural compounds that are receiving an increasing interest we can find the terpenylpurines and the terpenilquinones from marine sources [139,140]. Particularly, the terpenylquinones constitute an interesting group of marine natural product [141,142,143] for which a wide variety of biological activities have been described, including anti-inflamatory, antifungal, anti-HIV and most often antitumor activities [144,145]. 

**Figure 1:**
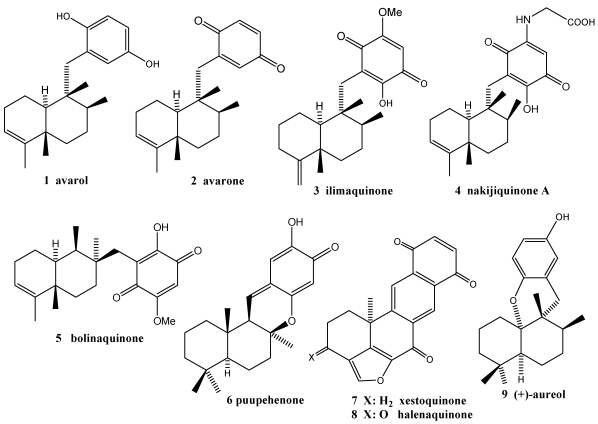
Some examples of bioactive terpenequinones/hydroquinones.

The cytotoxic and antiproliferative properties of many natural sesquiterpene quinones and hydroquinones from sponges of the order Dictyoceratida [71,76,140,144] such as avarol **1**, avarone **2**, illimaquinone **3**, nakijiquinone **4** and bolinaquinone **5** (Figure 1), amog others, offer promising opportunities for the development of new antitumor agents [144,145]. This has sparked interest in the chemical composition and cytotoxicity of a large number of marine species that contain metabolites with hybrid structures between terpenes and quinones/hydroquinones [76,140,141,146,147,148,149,150].

Avarol **1** and avarone **2** are the most representative compounds of bioactive terpenequinones. In addition to the above-mentioned pharmacological properties, two monophenyl thioavarol derivatives have recently been described as lacking cytotoxicity, which could point to promising UVB photoprotective agents through the potent inhibition of NF-kappaB activation [151] with a mild antioxidant pharmacological profile. Various formulations with high avarol **1** content have been used for the prevention and treatment of psoriasis, dermatitis, skin cancer, tumors in the gastrointestinal tract, urinary tract and viral infection [152].

It is also important to note the antituberculosis and antimalarial activities of puupehenone **6** [93,153,154], the cardiotonic activity of xestoquinone **7** [155], the antifungal activity of several nakijiquinones **4** [156] and the antiinfective activity of aureol derivatives **9** [157].

**Figure 2:**
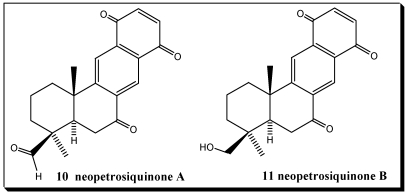
Neopetrosiquinones A **10** and B **11**.

Sesquiterpenequinones represent a substance class with increasing pharmacological interest [140]. New developments and new discoveries in the field of terpenequinones continually occur. Recently, neopetrosiquinones A **10** and B **11** (Figure 2), sesquiterpene benzoquinones have been isolated from the deep-water sponge *Neopetrosia* cf. *proxima*, of the Petrosiidae family [158]. Neopetrosiquinones A **10** and B **11** inhibit the *in vitro* proliferation of the DLD-1 human colorectal adenocarcinoma cell line with IC_50_ values of 3.7 and 9.8 μM, respectively, and the PANC-1 human pancreatic carcinoma cell line with IC_50_ values of 6.1 and 13.8 μM, respectively. Neopetrosiquinone A also inhibited the *in vitro* proliferation of the AsPC-1 human pancreatic carcinoma cell line with an IC_50_ value of 6.1 μM. The compounds are structurally related to known terpene quinine xestoquinone **7**. This research is part of the program to identify novel marine natural products with therapeutic properties from a library of extracts of the Harbor Branch Oceanographic Institute (HBOI) [158].

Regarding the mechanism of action of terpenylquinones, the accumulated data about the biological activity of quinone moieties suggest redox processes and/or Michael-type addition-elimination reactions [144]. Their cytotoxicity has been explained in terms of their ability to undergo redox cycling and the generation of reactive oxygen species, which would damage tumor cells [159,160,161]. NADH/NAD^+^ dehydrogenase reduction of the several terpenylnaphthoquinones increases the rate of oxygen consumption, such rates being higher for quinones with more positive redox potentials. In this process, reactive oxygen species are formed in small amounts, which also correlate with the quinine redox potential. Semiquinone derivatives of these quinones are generated under anaerobic conditions and in the presence of NADH/NAD^+^ dehydrogenase. Since this enzymatic system is found in mitochondria, a possible pathway in the cytotoxic activity of these terpenylnaphthoquinones could be by interference with or the inhibition of mitochondrial respiration, as reported for other naphthoquinone derivatives, in addition to free radical degradation [162,163]. The results obtained with avarol **1** and avarone **2** supported the mechanism of antitumor action via the reactive oxygen radicals [164,165] but there were also indications of the relevance of arylation of biomolecules, such as proteins [144,166,167].

Regarding such terpenequinone structures, many studies have been published addressing the isolation, structural elucidation, activity and mechanisms of action of the compounds [140,143,144,146,147,160]. We present in this review a compilation of the different synthetic approches for the preparation of the most relevant compounds.

## 2. Synthetic Approches Terpenylquinone/Hydroquinone

The synthesis of marine natural products has been widely researched and published in excellent reviews [3,168,169,170,171,172,173,174,175,176,177,178,179,180,181,182,183], and is of particular interest in the case of compounds that have some kind of biological or therapeutic activity. The two major obstacles to advancing a natural product lead into drug development are compound supply and adequate structural elucidation. One must not underestimate how much material may be needed. Even the most straightforward courses of pre-clinical studies require hundreds of grams of highly consistent well-characterized product, which represents a major hurdle for natural products derived from non-renewable sources [3]. Therefore, it is of interest to consider the relative role of chemical synthesis in the structure elucidation. Moreover, in the case of revision of relative and absolute configuration, total synthesis is a proven partner for natural product structure elucidation for marine, as well as terrestrial species [169]. Structural misassignments continue to be made even for recently reported marine natural products, and thus, it seems that the increasingly high-field magnets and sensitive probes do not necessarily attenuate the rate of structural misassignments. Rather, they permit the attempted structure elucidation of increasingly limited quantities of minor components from natural products extracts, as well as larger molecules of greater structural complexity. Therefore, total synthesis of natural products will surely continue to be central to the confirmation of the structure of natural products, as well as providing material for biological testing towards pharmaceutical development, and investigations of biosynthetic pathways [169]. Advances in total synthesis, especially function-oriented syntheses, biosynthetic technologies and genomic research offer new strategies for the medicinal chemical optimization of biologically active secondary metabolites as sources of novel drug leads [3].

In the case of biologically active terpenoquinones, the limited quantities components from the natural sources and the structural complexity are the main problems in continuining the clinical studies. Most of these terpenoquinones are characterised by possessing a quinone fragment attached to a terpenoid, which usually includes a decaline core, mostly with a drimane or rearranged drimane skeleton. Most sesquiterpenequinone/hydroquinones have been isolated from sponges, although some of them have been described from brown algae [74] and fungi [184]. The initial extract of the natural material usually consists of a complex mixture after fractionation. It may contain small quantities of bioactive substances, often as a mixture with structurally related molecules. The initial concentration of an interesting compound may be too low to be effectively tested in some biological and pharmacological assays. Thus, compounds have become attractive to carry out its total synthesis and obtaining of derivatives to improve the biological properties of natural compounds. Consequently, the development of these marine natural products is highly desirable and worthwhile from the viewpoint of medicinal chemistry and pharmaceuticals. 

In the present paper, the most interesting strategies addressed in the total synthesis of sesquiterpenilquinones by terpenic structure coupling to an aromatic ring have been reviewed. In general, the strategies employed in the total synthesis of sesquiterpenilquinones, are as follows:

➢ Diels-Alder cycloaddition reaction.
➢ Coupling of the aldehydes with lithiated hydroquinone ether.
➢ Radical decarboxylation and quinone addition reaction
➢ Grignard reagent conjugated addition to α,β-unsaturated carbonyl group.
➢ Reductive alkylation of enones.
➢ Cross-coupling reaction.
➢ Furylation of quinones.
➢ Furan polyene cationic cyclization.

In addition, the application of cell culture for the production of bioactive compounds from sponges is a promising way to utilize the bioactive potential of marine terpenoquinones sources.

## 3. Diels-Alder Cycloaddition Reaction

The Diels-Alder cycloaddition reaction continues to fuel the imaginations of synthetic chemists engaged in the assembly of complex molecular structures, in particular biologically significant natural products and provide rich opportunities for the rapid and selective generation of molecular complexity [185]. 

**Scheme 1:**
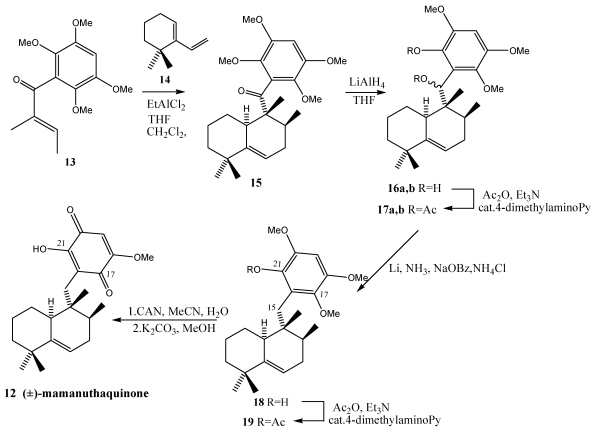
Synthesis of (±)-mamanuthaquinone **12**.

Mamanuthaquinone **12** is a cytotoxic metabolite collected in the Fiji islands from *Fasciospongia* sp. [186]. In the first total synthesis of (±)-mamanuthaquinone **12** (Scheme 1) [187] an exothermic Diels-Alder reaction have been used as main reaction, giving the decalin system that already contains the aromatic moiety. The Diels-Alder reaction proceeded via an *exo* transition state, favored by the steric hindrance between the aromatic ring of **13** and a methyl of the cyclohexene **14**. This *exo* approach mode reached the desired stereochemistry for mamanuthaquinone 12. With the configuration of the three stereocenters established, the cycloadduct **15** was treated with LiAlH_4_ yielding the reduction of the ketone and the demethylation of one methoxyl in the *ortho* position: diastereomers **16a,b**. The acetylated derivative **17a,b** which Li/NH_3_ gave deoxygenation at C-15 and was acetylated in alcohol C-21 leading to **19**. Finally, oxidation with CAN, followed by saponification of the acetate at C-21 yielded (±)-mamanutaquinone **12**.

**Scheme 2:**
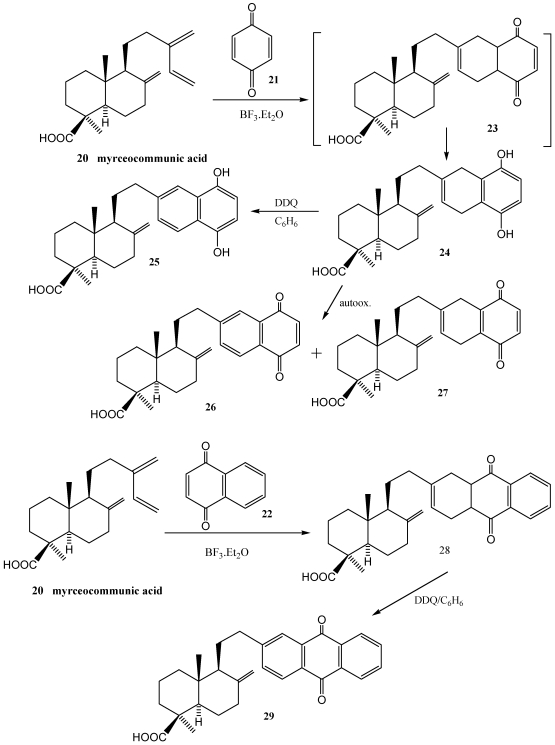
Diels-Alder cycloaddition between myrceocummunic acid and *p-*benzoquinone/naphthoquinone.

Starting from natural terpenes, various approaches to terpenoquinones analogues have been reported. The terpene contributes the decalin part that attaches via Diels-Alder cycloaddition to commercial quinones. Thus, some diterpenylquinones/hydroquinones have been prepared through a Diels-Alder cycloaddition between myrceocommunic acid **20** and *p*-benzoquinone **21** or naphthoquinone **22** [149,159,188] (Scheme 2). The natural labdane acid used as starting material was isolated from berries of *Juniperus oxycedrus*. In order to optimise the synthesis of cycloadduct, two Diels-Alder procedures were considered, one in ethereal solution using BF_3_·Et_2_O as a catalyst and the other under Mw irradiation in the absence of solvent. Although the Mw irradiation has the advantage of shortening reaction time, this procedure needs an excess of quinone that impeeds the purification of the final product. Several derivatives of cycloadducts **23** and **29** were evaluated *in vitro* for determinig their cytotoxicity against the human cell lines HT-29 (colon carcinoma), A-549 (lung carcinoma) and MEL-28 (malignant melanoma). Some of them were cytotoxic with IC_50_ values under the μM level.

**Scheme 3:**
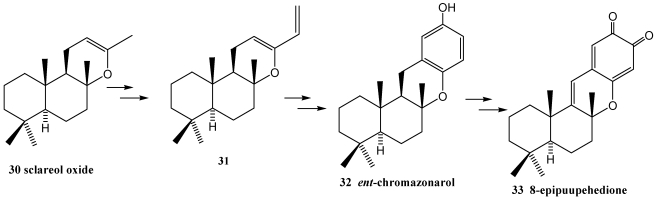
Diels-Alder cyclooaddition approach to puupehenone-related metabolites.

**Scheme 4:**
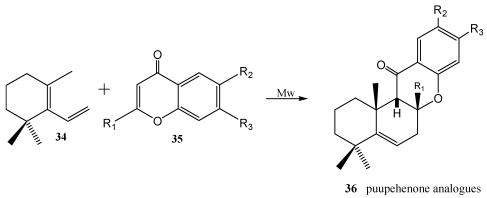
Microwave-assisted Diels-Alder reaction of 1,3,3-trimethyl-2-vinyl-1-cyclohexene with chromones.

Puupehenones belong to an important class of marine terpenequinone metabolites from *Hyrtios* sp. and other marine sponges [189,190,191,192], which are constructed from drimane and polyphenolic moieties. Puupehenones exhibit a wide variety of biological activity including angiogenesis inhibition [193]. The Diels Alder cycloaddition approach has been used to synthesize puupehenone related metabolites [194,195]. Utilizing this, the potent angiogenesis inhibitor 8-epipuupehedione **33** was synthesized from sclareol oxide **30**, via *ent*-chromazonarol **32** (Scheme 3); in this case, the methodologie used prevents the obtention of the 8-epimer which is formed when the electrophilic cyclization methodology is utilized [196,197]. Microwave-assisted Diels-Alder reaction of 1,3,3-trimethyl-2-vinyl-1-cyclohexene **34** with chromones **35** (Scheme 4) is an expeditious approach to analogues of the puupehenone group **36** of marine diterpenoids [198].

**Scheme 5:**
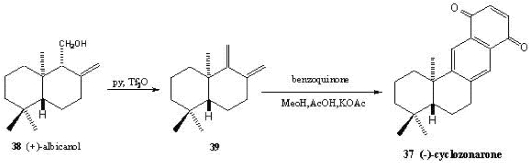
Synthesis of (−)-cyclozonarone **37**.

The marine (−)-cyclozonarone **37** has been isolated from the Pacific brown algae *Dictyopteris undulata* and possesses a potent feeding-deterrent activity towards young abalones [199]. The total synthesis was achieved starting from albicanol **38** (Scheme 5) [200]. Elimination of water led to drima-(8,12)(9,11)-diene **39**, which reacted in the key step of the synthesis, a Diels-Alder reaction, with benzoquinone. Further oxidation led to **37**.

**Scheme 6:**
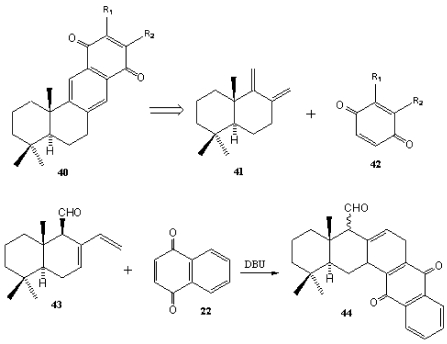
Diels-Alder reaction between polygodial-derived dienes and simple quinones.

The Diels-Alder cycloaddition between two polygodial-derived dienes **41** and **43** and simple quinones **42** and **22** yield substituted naphthaquinones **40** and anthraquinones **44**, some of them with *in vitro* trypanocide activity (Scheme 6) [201].

Halenaquinone **8**, a pentacyclic polyketide isolated from *Xestospongia* sp. [202], has been synthesized both through a strategy based on an intramolecular inverse-electron-demand Diels-Alder reaction and an intramolecular Heck cyclization [203,204].

## 4. Coupling of the Aldehydes with Lithiated Hydroquinone Ethers

This strategy is an efficient and general way of accessing drimane-type sequiterpenequinones. The strategy consists on the coupling of an aldehyde as terpene precursor and a lithium anion which carries the quinone structure. The five marine natural products yahazunol **45**, zonarone **46**, zonarol **47**, isozonarone **48** and isozonarol **49** have been synthesised starting from (+)-albicanic acid (+)-**51** [205,206]. Yahazunol **45**, zonarone **46** and zonarol **47** have been obtained from the East Pacific brown algae *Dictyopteris undulata* Okamura. Isozonarone **48** and isozonarol **49** have been isolated from the same species collected in the Gulf of California [207]. These compound present fungitoxic, anti-inflammatory activities and locks the MCF-7 cells initially in the mitose phase (G2/M-phase) and induce apoptosis, also blocks the synthesis phase with replication of DNA (S-phase) [72,142,207,208].

**Scheme 7:**
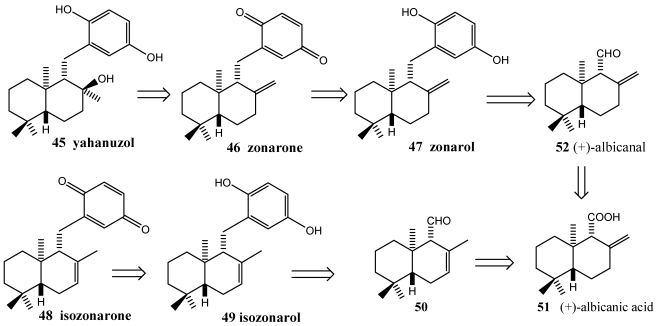
Retrosynthesis of yahazunol **45** and isozonarone **48**.

**Scheme 8:**
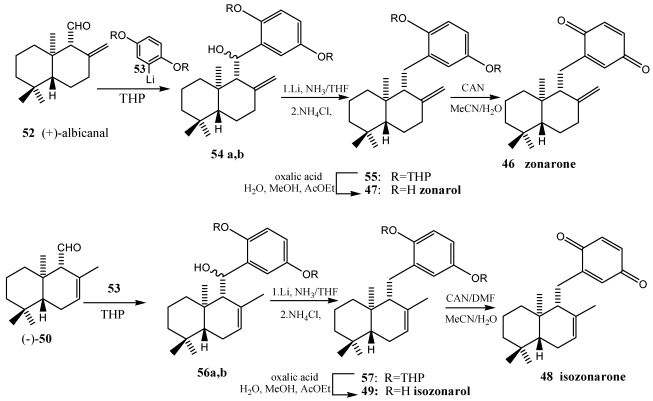
Total synthesis of zonarol **47**, isozonarol **49**, zonarone **46** and isozonarone **48**.

The synthesis of the marine natural products zonarone **46** and isozonarone **48** was achieved via (+)-albicanic acid **51**, a sesquiterpene of the drimane type (Scheme 7). Coupling of the appropiate drimane-synthon with lithiated hydroquinone ethers led to sesquiterpene arenes, which were further modified to the target molecules. Stereoselective epoxidation followed by regioselective opening of the oxirane ring yielded yahazunol [205,206]. The key step of the synthesis (Scheme 8) was the coupling of the sesquiterpene part with the lithiated arene unit. The starting quiral aldehydes (+)-albicanal ((+)-**52**) and (−)-drim-7-en-11-al (−)-**50** were obtained from (+)-albicanic acid (+)-**51**. This chiral synthon was prepared starting from β-ionone via a known route [209]. The di-THP-ether of hydroquine was lithiated with *sec*-butyllithium and added (+)-albicanal **52**, respectively (−)-drim-7-en-11-al **50**, to formed lithium organyl. The reaction afforded the benzyl alcohol **54a,b** and **56a,b**, as coupling products as mixture of diastereoisomers. Removal of the hydroxyl group led to the deoxygenated species zonarol-di-THP-ether **55** and isozonarol-di-THP-ether **57**, respectively. Desprotection in presence of oxalic acid [205] gave zonarol **47** and isozonarol **49**. Optimized oxidation of zonarol and isozonarol with cerium (IV) ammonium nitrate (CAN) yielded the sesquiterpenequinone zonarone **46** and isozonarone **48**.

**Scheme 9:**
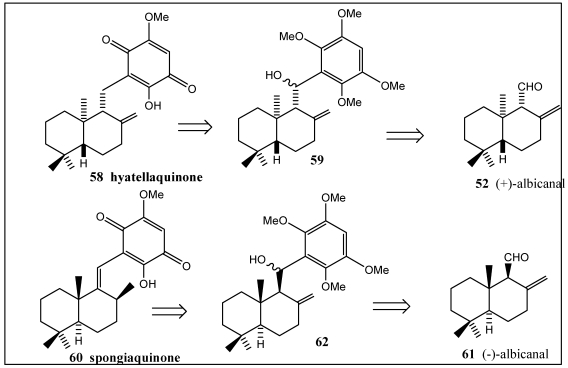
Retrosynthesis of hyatellaquinone **58** and spongiaquinone **60**.

The synthesis of the marine sesquiterpene quinones (+)-hyatellaquinone **58** and spongiaquinone **60** was respectively achieved starting from the sesquiterpene aldehydes (+)-albicanal **52** and (−)-albicanal **61** (Scheme 9) [210,211]. The sesquiterpene quinone hyatellaquinone has been isolated from the alga *Peyssonnelia* sp. and the marine sponges *Hyatella intestinalis* [212] and *Spongia* sp. [211]. Spongiaquinone **60** has been obtained from the sponges *Spongia* sp. [211] and *Stelospongia conulata* [213]. These terpenequinone were attractive candidates for pharmacological testing as antitumor, HIV-1 reverse transcriptase inhibitor and immunomodulatory activities [72,142,208].

**Scheme 10:**
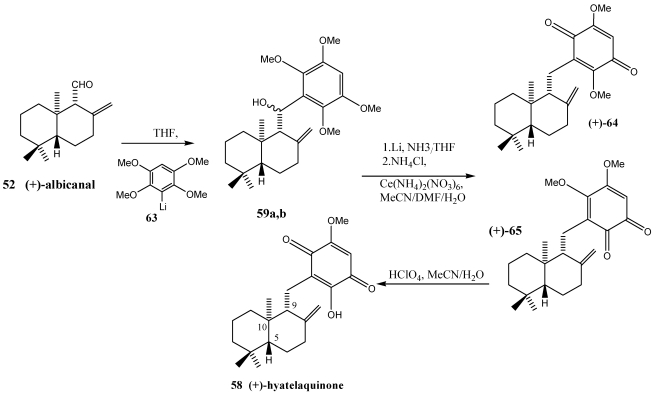
Synthesi of (+)-hyatellaquinone **58**.

**Scheme 11:**
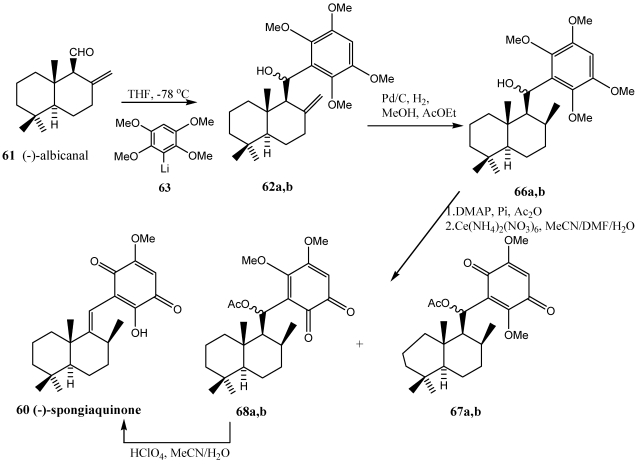
Synthesis of (−)-spongiaquinone **60**.

The synthesis of (+)-hyatellaquinone **58** was achieved starting from the sesquiterpene aldehyde (+)-albicanal **52** (Scheme 10) [210]. Coupling of (+)-albicanal **52** with 2,3,5,6-tetramethoxyphenyllithium **63** led to the aryl-sesquiterpene system **59**, which was modified to the target molecule. Furthermore, the first total synthesis of spongiaquinone **60** was carried out starting from (−)-albicanal **61** (Scheme 11) [211] in a reaction sequence encompassing a stereoselective C=C bond hydrogenation and a one-pot AcOH elimination/demethylation reaction.

**Scheme 12:**
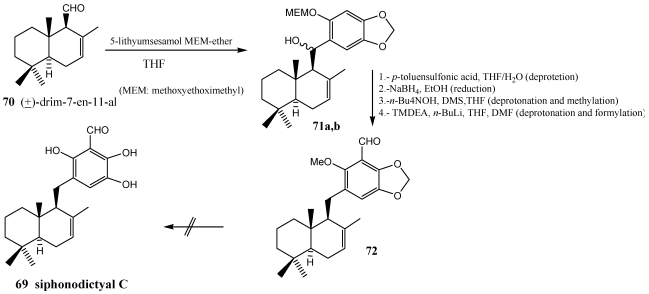
Synthesis of protected siphonodictyal C **69** via drim-7-en-11-al **70**.

Siphonodictyal C **69**, isolated from sponge *Siphonodictyon* sp. [214,215], was tested for its pharmacological activities in assays in search of antiproliferative, cytotoxic, antiphlogistic, antirheumatic and anti-inflammatory drugs [70,208]. Synthesis of siphonodictyal C 69 was achieved via drim-7-en-11-al **70** by coupling with 5-lithium sesamol MEM-ether to the benzylic alcohols (±)-**71a,b** (Scheme 12) [206,208]. Treatment of (±)-**71a,b** with *p*-toluene sulfonic acid (PTS) in THF/H_2_O led to the deprotection of the MEM-group and benzylic dehydration. The formed phenol was rearranged in a six membered cyclic transition state to the alkylidenecyclohexadienone which by reduction with NaBH_4_ in EtOH yielded the phenol that was deprotonated with *n*-Bu_4_NOH and the phenolate was methylated with dimethylsulfate (DMS), deprotonated with *n*-BuLi in o-position to the methoxy-group and formylated with DMF to (±)-**72**. The deprotection of (±)-**72** with different reagents always led to decomposition. 

**Figure 3:**
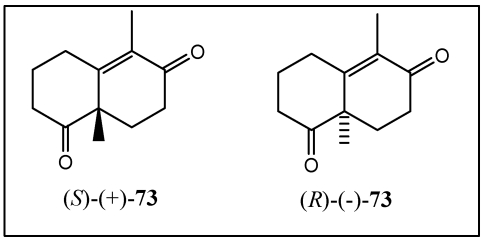
Wieland-Miescher Ketone **73**.

**Scheme 13:**
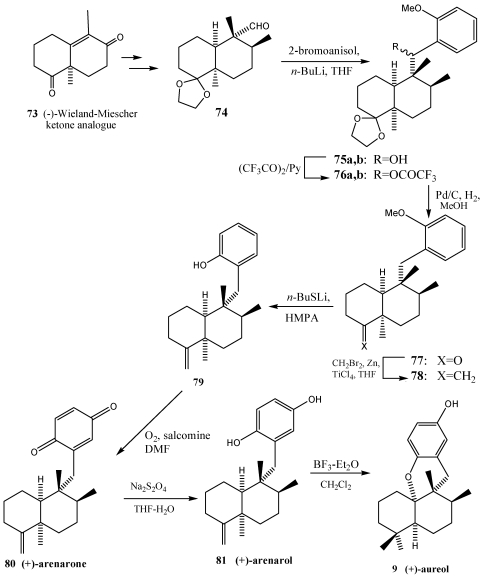
Synthesis of (+)-arearone **80**, (+)-arenarol **81** and (+)-aureol **9** staning from *cis*-fused decalin.

In addition, aureol **9** and their analogues were synthesized by coupling of the aldehydes with lithiated hydroquinone ethers using, in this case, a *cis*-decaline as starting material [216,217]. Aureol was isolated from the Caribbean sponges *Smenospongia aurea* [218] and *Verongula gigantea* [219]. Aureol **9** has been shown to exhibit selective cytotoxicity against A-549 human non-small cell lung cancer cells and antiinfluenza-A virus activity [67,220]. As shown in Scheme 13, the synthesis commenced with the crucial coupling reaction of the *cis*-fused aldehyde [221,222] previously prepared from the enantiomerically pure (−)-Wieland-Miescher ketone **73** analogue (Figure 3) [223,224] with commercially available 2-bromoanisole. 

Thus, the aryllithium generated *in situ* by treatment of 2-bromoanisole with *n*-butyllithium in THF was allowed to react with **74** providing an excellent yield of the desired coupling product **75**. Simultaneous removal of both the benzylic hydroxyl group and the ethylene acetal moiety in **75** was achieved effectively by initial formation of the corresponding trifluoroacetate **76** followed by reaction under the conditions for hydrogenolysis, wich led to the production of the carbonyl group **77**. Subsequent methylenation of the sterically hindered carbony group in **77** was achieved by employing the Takai procedure [225]. Thus, treatment of **77** with a mixture of dibromoethane, zinc powder and titanium (IV) chloride in THF furnished the *exo*-olefinic compound **78**. The methylenation of **77** with Wittig reagent, Peterson’s reagent or Tebbe reagent gave none of desired product **78**. Next, deprotection of the methyl ether protecting group of *exo*-olefin by treatment with lithium *n*-butylthiolate in hexamethyl-phosphoramide afforded the liberated phenolic compound **79**. The pivotal conversion of the phenolic derivative to arenarone **80** was effected by reaction of **79** with molecular oxygen in the presence of salcomine in DMF. Subsequent reduction of the quinone system in arenarone **80** using sodium hydrosulfite gave arenarol **81**. By treating of arenarol with BF_3_·Et_2_O, the desired acid-promoted rearrangement/cyclation reaction was found to proceed, producing aureole **9** with a good stereoselectively in excellent yield [221,222].

**Scheme 14:**
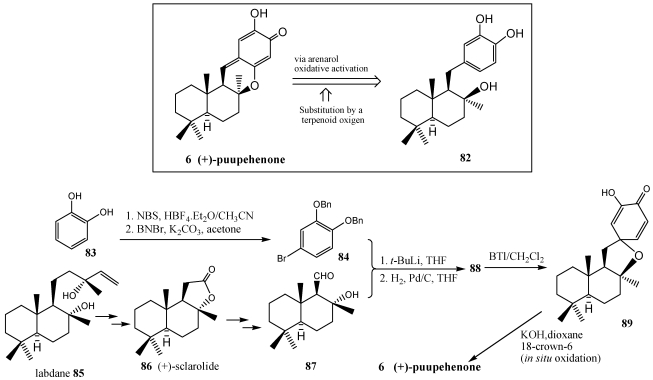
Enantiospecific synthesis of (+)-puupehenone **6**. The arenol oxidative activation route.

The total synthesis of the (+)-puupehenone **6** was achieved in 10 steps (Scheme 14) by the arenol oxidative activation route [153] starting from commercially available (+)-sclareolide **86**. The key feature of this synthesis is the construction of the heterocycle via an intramolecular attack of the terpenoid-derived C-8 oxygen function onto an oxidatively activated 1,2-dihydroxyphenyl unit. The sequiterpene moiety of puupehenone **6** features a normal drimane skeleton annelated to a shikimate-derived hydroxyquinone unit. The drimane precursor (+)-sclarolide **86** already possesses the correct chirality for three of the four (+)-puupehenone **6** stereogenic centers. It can be purchased from commercial sources or convenientelly prepared from labdane **85** [226]. The nucleophilic character of the terpenoid 8-oxygen will serve to mediate the desired heterocyclization by attacking an oxidatively activated 1,2-dihydroxyphenyl unit. The shikimate unit **84** was elaborated from catechol **83** through bromination and benzylation to give bromide. Coupling of this bromide with aldehyde **87** obtained from (+)-sclarolide **86**, was achieved via a standard halogen-metal exange protocol. A subsequent hydrogenolysis under standard conditions allowed removal of both benzyl protective groups, and the benzylic C-15 hydroxyl group that was unveiled at the previous coupling reaction, to afford the catechol **88** in good yield. The remarkable deprotection-deoxygenation step set the stage for the key oxidative activation of the catechol unit toward intramolecular attack by the drimane 8-oxygen. This activation relied on the use of [bis(trifluoroacetoxy)iodo]benzene (BTI), that as other iodine reagents, constitute today a convenient alternative to the use of toxic heavy metal-based reagents for activating arenols toward oxidative nucleophilic substitution reaction [227,228].

**Scheme 15:**
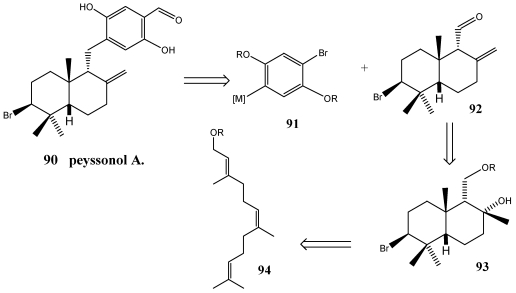
Retrosynthetic analysis of peyssonol A **90**.

The synthesis of peyssonol A **90** is a special case of fusion between a *cis*-decalin and the aryl ring [229]. Peissonol A was isolated from the Red Sea marine alga *Peyssonnelia* sp. that has been shown to act as an allosteric inhibitor of the reverse transcriptases of Human Immunodeficiency Virus [212,230]. This compound is the only known natural product possessing a *cis*-decalin framework likely arising from a halonium-induced cation-π cyclization. As indicated in Scheme 15, the retrosynthetic analysis suggested that the late-stage disconnection on the pendant aryl ring projecting a nucleophilic addition onto the aldehyde **92** to effect its incorporation, might afford the most efficient means to reach a suitable polyene cyclization precursor.

## 5. Radical Decarboxylation and Quinone Addition Reaction

The application of the Barton’s radical decarboxylation reaction, in which the generated radicals are trapped by a quinone trap, gives rise to addition products in good to excellent yields. This addition reaction is characterized by good chemoselectivity, taking place only at conjugated and unsubstituted double bonds, and regioselectivity, being strongly influenced by the resonance effect of heteroatoms located on the quinone ring. The synthetic value of this reaction was demonstrated by the synthesis of selected members of a family of quinone sesquiterpenes. Both symmetric and unsymmetric quinones can be used as radical traps and provide facile access to heteroatom-substituted quinone sesquiterpenes. The versatility of our strategy was further expanded by developing reaction conditions that allow subsequent oxygenation of the quinone adducts, providing access to complementary oxygenated structures [231]. 

**Scheme 16:**
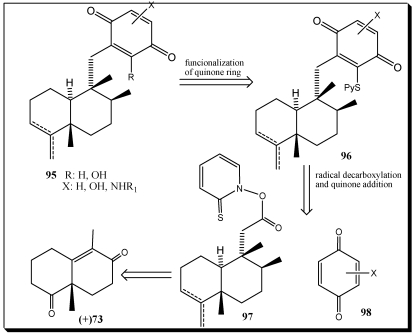
Strategic bond disconnections of quinone sesquiterpenes.

Essential to this strategy is a radical addition reaction that permits the attachment of a fully substituted bicyclic core **97** to a variably substituted *p*-quinone **98** (Scheme 16). The addition product **96** can be further functionalized, giving access to natural products with a high degree of oxygenation at the quinone unit. The quinone addition reaction is characterized by excellent chemoselectivity, taking place only at conjugated and unsubstituted double bonds, and regioselectivity, being strongly influenced by the resonance effect of heteroatoms located on the quinone ring. These features were successfully applied to the synthesis of avarol **1**, avarone **2**, ilimaquinone **3** and smenospongidine **116**, thereby demonstrating the synthetic value of this method [231].

Avarol **1** and its quinone derivative avarone **2** are secondary metabolites isolated from the marine sponge *Dysidea avara* [232,233]. Both compounds were first discovered as anti-leukaemia agents *in vitro* and *in vivo*, and later it was found that they had an *in vitro* inhibitory capacity against HIV-1. Controlled clinical studies revealed, however, that it was not efficient in the clinical treatment of patients with AIDS. Additionally, the potent T-lymphotropic cytostatic activity shown by avarol **1**, and its low toxicity in mice, its ability to cross the blood-brain barrier and its ability to stimulate the synthesis of interferon make both these compounds optimum candidates for transformations aimed at improving their cytostatic and antiviral activity [234,235,236,237,238]. 

**Scheme 17:**
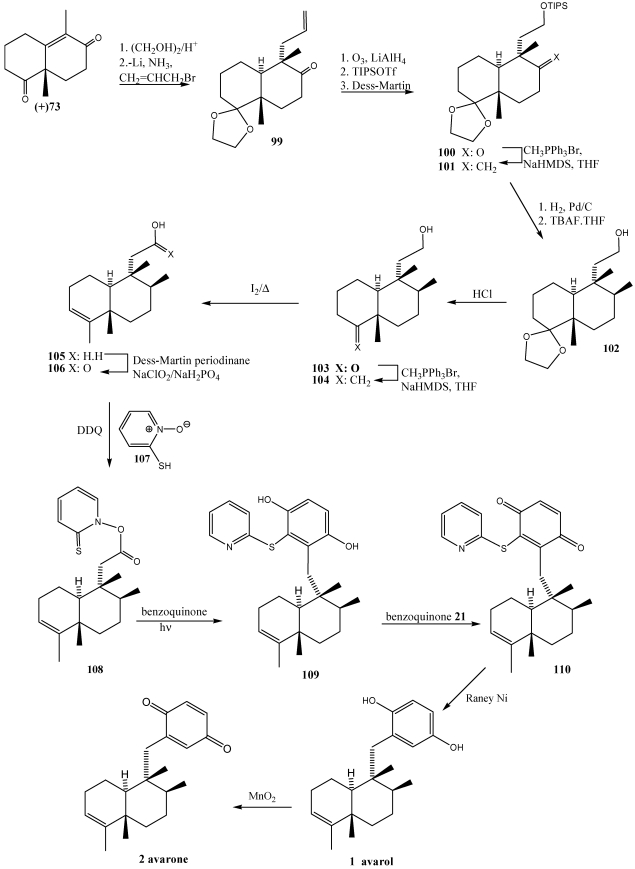
Total synthesis of avarol **1** and avarone **2** via radical decarboxyation and quinone addition reaction.

The synthetic approach toward the core fragment of avarol and avarone (Scheme 17) began with enantimerically enriched enone **73** (Figure 3), which was readily available through a L-phenylalanine-mediated asymmetric Robinson annulation [239]. The selective protection of the more basic C4 carbonyl group followed by reductive alkylation of the enone functionality with allyl bromide afforded ketone **99**. Conversion of ketone **99** to silyl ether **100** was accomplished via a sequence of three steps including ozonolysis of the terminal double bond, reduction of the resulting aldehyde, and selective silylation of the primary alcohol. The C8 ketone functionality that also suffered reduction during the above procedure was subsequently restored upon treatment with Dess-Martin periodinane [240]. Functionalization of C8 stereocenter was achieved by Wittig olefination, followed by a Pd-catalyzed hydrogenation of the resulting exocyclic methylene unit, furnishing alcohol **102**. Acid-catalyzed deprotection of the C4 ketal of **102** gave rise to ketone **103**, which a second Witting methylenation provided the exocyclic alkene **104**. The exocyclic double bond of **104** was isomerized to produce de most substituted alkene **105**, wich after a two-step oxidation involoving Dess-Martin periodinane and sodium chlorite, produced the desired carboxylic acid **106**. The stage was now set for the attachment of the aromatic residue on the decalin ring. This was accomplished by DCC-induced esterification of **106** with commercially available 2-mercaptopyridine-*N*-oxide **108**, which furnished the photolabile ester **108**. Light-induced decarboxylation of ester **108** in the presence of benzoquinone **21** produced the substituted quinone **110**. At this point, brief treatment of **110** with Raney nickel produced synthetic avarol **1** in 84% yield. Consequently, avarone **2** was produced from **1** via heterogeneous oxidation with MnO_2_.

**Scheme 18:**
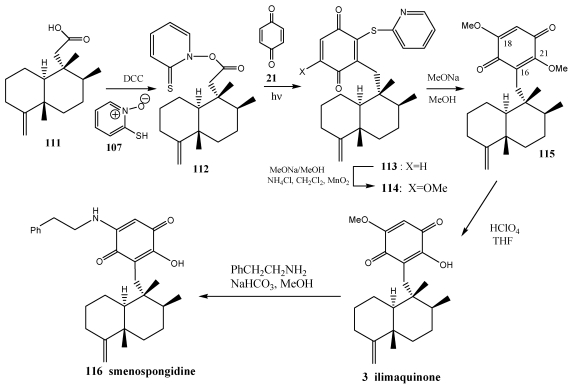
Total synthesis of ilimaquinone and smenospongidine.

One of the synthetic strategies to ilimaquinone **3** and smenospongidine **116** is also based on a radical decarboxylation and quinone addition methodology (Scheme 18) [231,241]. These terpenoquinones were colleted from *Hippospongia* sp. [224,242,243]. The cytotoxicity against the NCI-H460, HepG2, SF-268, MCF-7, HeLa, and HL-60 human tumour cell lines, the inhibitory effects on the maturation of starfish oocytes, and cell cycle arrest in the HepG2 cell line were evaluated [242].

The chemical structures of ilimaquinone **3** and smenospongidine **116** differ from those of avarone-like molecules at the position of unsaturation of the decalin core and the additional oxygenation at the C21 center of the quinone ring. The application of radical decarboxylation and quinone addition methodology produces quinone **113** from reaction of thiohydroxamic acid derivative with benzoquinone **21**. Functionalization of **113** to ilimaquinone **3** is achieved by exploiting the electronic effects of the residual thiopyridyl group. Finally, exposure of **3** to phenylethylamine under basic conditions afforded synthetic smenospongidine **116**.

**Scheme 19:**
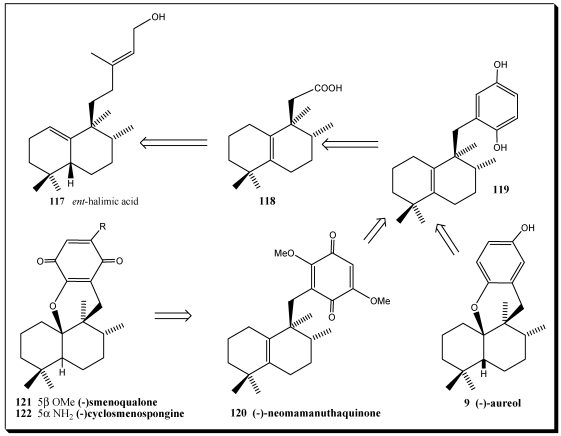
Retrosynthetic analysis of some sesquiterpenequinones/hydroquinones from *ent*-halimic acid.

*Ent*-halimic acid **117** is used as starting material for the synthesis of aureole (−)-**9**, neomammanuthaquinone **120**, smenoqualone **121**, and cyclosmenospongine **122**. The Barton decarboxylation in presence of benzoquinone is the key reaction in this synthesis (Scheme 19) [244].

## 6. Grignard Reagent Conjugated Addition to α,β-Unsaturated Carbonyl Group

**Scheme 20:**
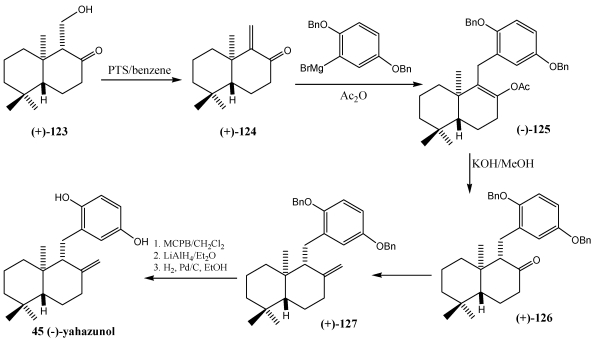
Synthesis of yahazunol **45** by Grignard reagent conjugated 1,4-addition to enone 12-nordrim-9-dn-8-one.

**Scheme 21:**
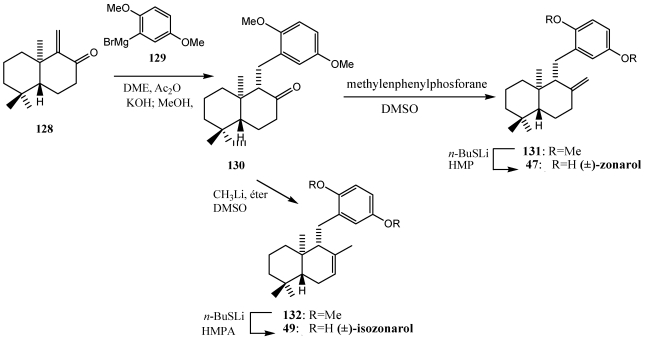
Synthesis of (±)-zonarol **47** and (±)-isozonarol **49** by Grigard reagent conjugated 1,4-addition.

The total synthesis of (±)-zonarol **47**, (±)-isozonarol **49** [205,206] and (−)-yahazunol **45** [207,208] also were achieved by Grignard reagent (1,4) conjugated addition to α,β-unsaturated carbonyl group (Scheme 20 and Scheme 21) [245].

The synthesis of yahazunol **45** started from (+)-11-hydroxy-12-nordriman-8-one **123** which was transformed with *p*-toluene sulfonic acid by elimination of water to enone (+)-**124**. The cuprate catalyzed conjugated 1,4-addition of 2,4-dibenzyloxyphenylmagnesium bromide to (+)-12-nordrim-9-en-8-one (+)-**124** yielded the enolate anion which was trappe with acetic anhydride. Treatment of the resulting enolacetate (−)-**125** with potassium hydroxide in methanol afforded the ketone (+)-**126**. Wittig reaction of (+)-**126** with PH_3_PCH_2_ gave (+)-zonarol dibenzyl ether (+)-**127**. Epoxidation in position 8–12 gave the oxirane ring which was opened with LiAlH_4_ to (+)-yahazunol dibenzyl ether. Debenzylation of (+)-10 with H_2_ and Pd/C yielded (−)-yahazunol **45** (Scheme 20) [245].

In the synthesis of (±)-zonarol **47** and (±)-isozonarol **49** (Scheme 21), the terpene ketone **128**, was prepared following a sequence of reactions [246] from the racemic mixture of the Wieland-Miescher ketone **73** (Figure 3). This ketone and its analogs are of great interest as starting materials to terpenequinones by asymmetric synthesis [223,224,239]. After preparing the Grignard reagent **129**, which provides the hydroquinone moiety, it was reacted with the α,β-unsaturated ketone **128**, and Ac_2_O, yielding the enol acetate obtained by conjugate addition. Treatment of the enol acetate with KOH gave ketone **130**, which established the stereochemistry of the molecule. From compound 130, (±)-zonarol **47** and (±)-isozonarol **49**, were obtained by two different ways. Wittig reaction, followed by demethylation, zonarol racemate **6** was obtained. By 1,2-addition of organolithium to the ketone **130**, (±)-isozonarol **49** was obtained through a tertiary alcohol, which by dehydration gave a mixture of compounds **131** and **132**. Finally, the demethylation of the methoxy group by treatment with lithium butanetiolate and HMPA led to (±)-zonarol **47** and (±)-isozonarol **49**.

## 7. Reductive Alkylation of Enones

This strategy to connect the unit to a terpene quinone is based on the alkylation in the reaction medium during metal reduction of a conjugated double bond. Lithium with a solvent proton reduces the double bond through electron transfer giving an enolate. Thus, alkyl halide reaction generates the desired alkylated ketone. In all cases, the α,β-unsaturated ketone that is coupled to the quinone has the (*S*)-(+)-Wieland-Miescher diketone **73** as the starting material. 

**Scheme 22:**
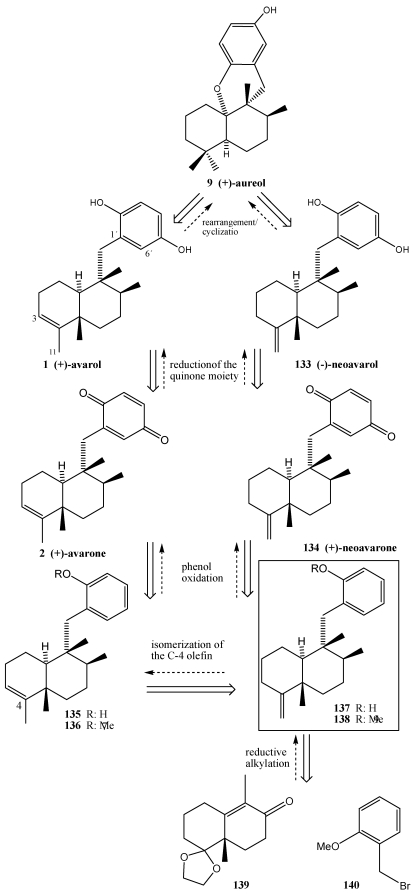
Synthetic plan for (+)-avarone **2**, (+)-avarol **1**, (−)-neoavarone **134**, (−)-neovarol **133** and (+)-aureol **9** by enones reductive alkylation.

The synthesis of (+)-avarone **2**, (+)-avarol **1**, (−)-neoavarone, **134** (−)-neovarol **133** and (+)-aureol **9** is a good example of reductive alkylation of enones with bromides (Scheme 22) [247]. Thus, the enone **139** with bromide **140**, and applying previously described protocols from the literature [156,239,248,249,250,251] gave the *exo*-olefin **138**. The *endo*-olefin **136** should be accessible from **138** by isomerization at the C4 olefinic double bond.

**Scheme 23:**
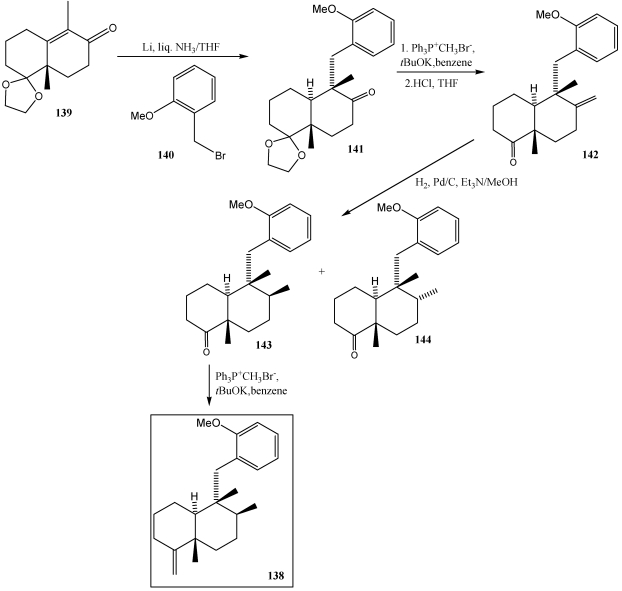
Synthesys of key intermediate **138**.

The synthesis of the decalin derivative **138** (Scheme 23), a common key intermediate for the synthesis of (+)-avarone **2**, (+)-avarol **1**, (−)-neoavarone **134**, (−)-neovarol **133** and (+)-aureol **9**, started with the reductive alkylation of enantiomerically pure enone **139** with 2-methoxybromide **140**. Thus, treatment of enone **139** with lithium metal in liquid ammonia followed by reaction of the intermediary lithium enolate with bromide **140** provided the expected coupling product **141** as a simple diastereomer. Subsequent methylenation of the sterically hindered carbonyl group in **141** was achieved by employing a combination of Ph_3_P+CH_3_Br- and *t-*BuOK furnishing the *exo*-olefin. To establish the C8 sterogenic center, the ethylene acetal moiety was first removed by acid treatment and the resulting ketone **142** was subjected to hydrogenation, which afforded the product **143** and its C8 epimer **144** after separation by coumn chromatography on silica gel. Finally, compound **144** was efficiently converted to the desired key intermediate **138** by Wittig methylenation [247].

**Scheme 24:**
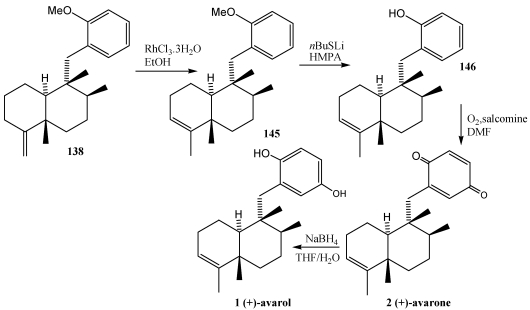
Synthesis of avarol **1** and avarone **2** reductive alkylation of enones.

The Scheme 24 shows the synthesis of avarol **1** and avarone **2** from key intermediate **138**. Isomerization of the *exo*-olefin moiety in **138** to *endo*-olefinic double bond was efficienthly achieved by treatment with RhCl_3_·3H_2_O. The *endo*-olefin **145** was then converted to avarone **2** and avarol **1** via phenol **146**. To construct the quinone system directly, phenol 146 was allowed to react with molecular oxygen in the presence of salcomine, producing (+)-avarone **2**. Subsequent treatment of avarone **2** with NaBH_4_ in THF/H_2_O resulted in the quinol avarol **1** [247].

**Scheme 25:**
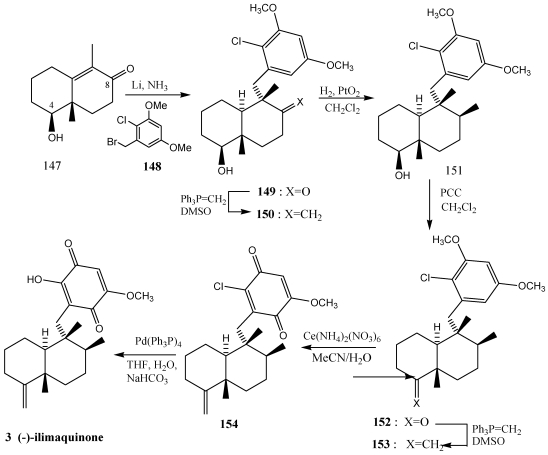
Synthesis of ilimaquinone **3** by reductive alkylation of enones.

Ilimaquinone 3 [224,242] and nakijiquinones **4, 163**–**165** [252,253,254,255,256,257] have also been synthesized using this strategy. In the synthesis of (−)-ilimaquinone **3** (Scheme 25) [250,251], compound **147** was treated with Li/NH_3_ giving the lithium enolate and subsequent treatment with benzyl halide **148** led to the α-alkylation product **149**. The configuration of the remaining stereocenter (C-8) was established with a Wittig reaction followed by hydrogenation to yield compound **151** and its diastereomers (3:1). The oxidation of alcohol at C-4 and subsequent formation of the olefin led to **153**. Finally, treatment with CAN and Pd (0) in basic medium, yielded (−)-ilimaquinone **3**.

**Scheme 26:**
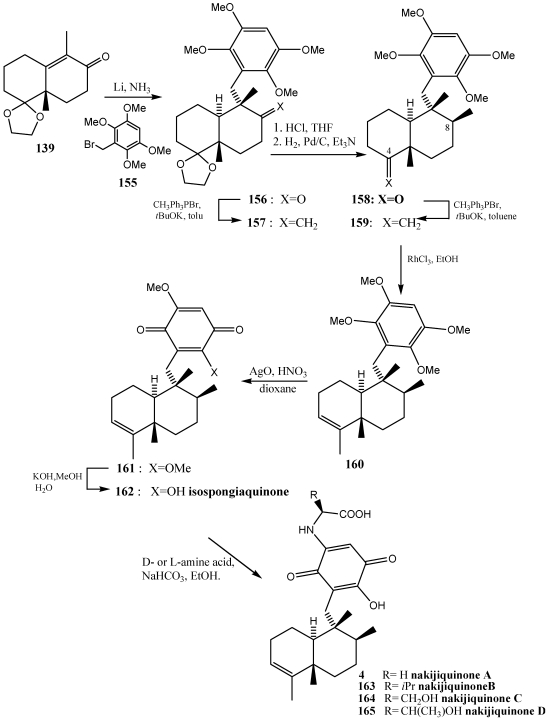
Synthesis of isospongiaquinone and nakijiquinone A–D **4**, **163**–**165** by reductive alkylation of enones.

The reductive alkylation as strategy for building sesquiterpenilquinones has also been used in the synthesis of nakijiquinones [156,252]. From extracts of sponges from the family Spongiidae, collected in Okinawa several nakijiquinones were isolated. Nakijikinones are terpenequinones with an amino acid on the benzoquinone ring [253,254,255,256,257]. The HER2/Neu receptor tyrosine kinase is hugely overexpressed in about 30% of primary breast, ovary, and gastric carcinomas. Nakijiquinones are the only naturally occurring inhibitors of this important oncogene, and structural analogues of nakijiquinones may display inhibitory properties against another tyrosine kinase receptor involved in cell signaling and proliferation [156]. The synthetic route (Scheme 26), was optimized to obtain nakijiquinone C, using as intermediate the isospongiaquinone **162** and later the strategy was extended to obtain nakijiquinones A–D **4, 163**–**165**.

## 8. Cross-Coupling Reaction

**Scheme 27:**
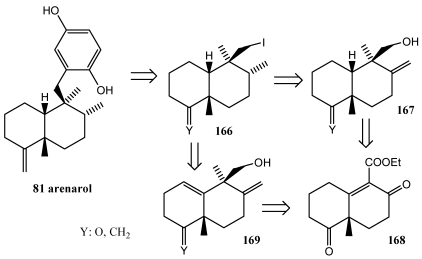
Retrosynthetic analysis of arenarol **81**.

This strategy consists on the application of a (dppf)NiCl_2_-mediated neopentyl coupling in natural product synthesis and emphasizes the attractive combination of hydroxyl-directed hydrogenation to control stereochemistry followed by a neopentyl coupling to elaborate the carbon skeleton. Retrosynthetic analysis as summarized in Scheme 27 readily dissects arenarol to a neopentyl iodide **166** and 2,4-dimethoxyphenylmagnesium bromide [258]. The neopentyl iodide turn could be derived from the corresponding alcohol **167**, assuming that the hydroxyl group could be employed to control the stereochemistry of reduction at an adjacent exocyclic olefin, or the diene alcohol **169**, if the hydroxyl group could be employed to fix both adjacent stereocenters. Either olefin could be viewed as a derivative of the decalin **168**, depending on the sequence employed to accomplish methylation, introduction of the exocyclic olefin, and for compound **168**, reduction of the endocyclic olefin. The synthesis of arenarol, based on this approach, includes both directed introduction of two key stereogenic centers and a (dppf)NiCl_2_-mediated coupling at a neopentyl center.

**Scheme 28:**
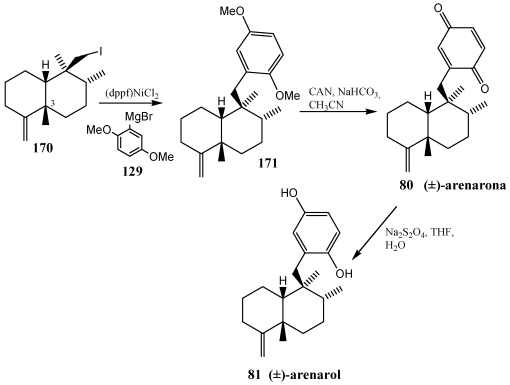
Synthesis of arenarone **80** and arenatol **81** by cross-coupling reaction.

Arenarol **81**, isolated from *Dysidea* sp. and *Fenestraspongia* sp. [259,260] is a *cis*-decalin the synthesis of which calls for stereocontrol at two tertiary and two quaternary carbons. These compounds showed cytotoxic activity when assayed against P-388 leukaemia cells, with ED_50_ = 17.5 μg/mL for arenarol **81** and ED_50_ = 1.7 μg/mL for arenarone **80** [259]. Arenarol **81** showed DPPH radical scavenging activity with an IC_50_ value of 19 μM [260]. The Grignard reagent needed for preparation of arenarol **81**, (2,5-dimethoxyphenyl)magnesium bromide **129**, has been shown to undergo a cross-coupling reaction with neopentyl iodide in the presence of (dppf)NiCl_2_ and Zn_2_ dioxane forming the desired coupling product **171**. Conversion of **171** to the target compounds required cleavage of the methyl protecting groups. Treatment of compound **171** with ceric ammonium nitrate (CAN) resulted in oxidation to the natural product arenarone **80**. Mild reduction of arenarone **80** with Na_2_S_2_O_4_ gave the final target arenarol **81** (Scheme 28).

## 9. Furylation of Quinones

**Scheme 29:**
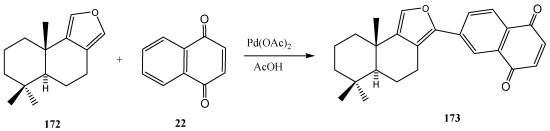
Furylation of quinones.

This procedure consists on furylation of quinones and hydroquinones through oxidative coupling and Michael addition reactions. Thus, the oxidative coupling reaction of (+) euryfuran with 1,4-quinones in acetic acid yielded euryfuryl-1,4-quinones with leishmanicidal activity. The influence of the solvent to promote the Michael addition and the regioselectivity of the reaction with unsymmetrical quinones are important feactures that can be useful for the synthesis of new bioactive members of the euryfurylquinones series [261] (Scheme 29). The Michael reaction of (+) euryfuran **172** with activated monosubstituted 1,4-benzoquinones **22** provides a regiospecific access to antiprotozoal active euryfuran derivatives **173** containinig a quinone or hydroquinone fragment bond to the 12 position [262]. Access to furylnaphthoquinones from unactivated quinones requires acid-induced conditions. However, oxidative coupling reactions of activate quinones proceed under neutral conditions. Most of the furyl-1,4-quinones exhibited good antiproliferative activity against MCF-7, NCI-H460 and SF-268 cancer cell lines [145].

## 10. Furan Polyene Cationic Cyclization

This strategy is a diversity-oriented synthesis that follows a biomimetic route [263] to marine natural products like liphagal **1**, the first member of a new of new liphagane type of meroterpenoid carbon skeleton. Liphagal **180** isolated from the methanol extract of the sponge *Aka coralliphaga*, collected from reefs in Prince Rupert Bay, Portsmouth, Dominica [264] exhibited impressive biological activity including inhibitory activity against PI3K a (phosphoinositide-3-kinase α) and cytotoxic to LoVo and CaCo human colon, and MDA-468 human breast tumor cell lines [264,265,266]. 

**Scheme 30:**
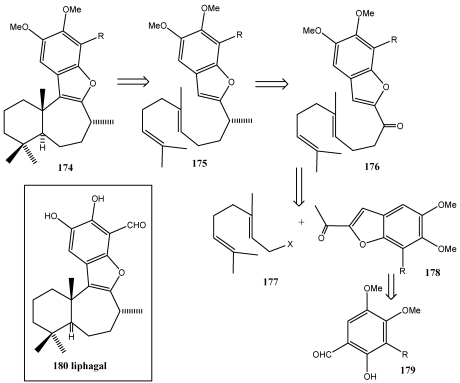
Synthesis of meroterpenoid natural product (±)-liphagal **180** Retrosynthetic analysis by furan polyene cationic cyclization.

Liphagal **180** has a tetracyclic skeleton, harboring a *trans*-fused 6,7-bicarbocyclic core with three stereogenic centers. The retrosynthetic strategy toward liphagal (Scheme 30) was based on the proposed biogenetic pathway and hinged on a key C–C bond disconnection that mandated connecting a preformed benzofuran precursor **178** with a readily available monoterpenoid **177** to establish the crucial C-C bond and access the framework 8. Further elaboration of **176** into **175** was envisaged to set up the furan polyene cationic cyclization cascade en route to the target.The key furan precursor **178** was to be ascessed from a readily available aromatic precursor **179**.

## 11. Application of Cell Culture for the Production of Bioactive Compounds from Sponges

Sponges [phylum Porifera] are a rich source of biologically active and pharmacologically valuable compounds with a high potential to become effective drugs for therapeutic use. However, until now, only a few compounds have been introduced into clinics because of the limited amounts of starting material available for extraction. To overcome this serious problem in line with the rules for a sustainable use of marine resources, the following routes can be pursued; first, chemical synthesis, second, cultivation of sponges in the sea (mariculture), third, growth of sponge specimens in a bioreactor, and fourth, cultivation of sponge cells *in vitro* in a bioreactor [267].

Recently, it was demonstrated that the *in vitro* culture of primmorph from the marine sponge *Dysidea avara* produces avarol **1**. Single cells apparently do not have the potency to produce this secondary metabolite, but the primmorph model is a suitable system for the synthesis of bioactive compounds *in vitro* [268,269]. In addition, it has also been suggested that some of the bioactive secondary metabolites isolated from sponges are produced by functional enzyme clusters, which originated from the sponges and their associated microorganisms. In order to exploit the bioactive potential of both the sponge and the “symbionts”, a 3D-aggregate primmorph culture system was studied, and it was proved that avarol/avarone is produced by the sponge *Dysidea avara.* Another promising way to utilize the bioactive potential of the microorganisms is the cloning and heterologous expression of enzymes involved in secondary metabolism [270].

*In situ* sponge aquaculture is nowadays one of the most reliable methods to supply pharmaceutical companies with sufficient quantities of the target compound. Its use in addition to immortalization of sponge cells by transfection with genomic DNA appears to be a promising way, since recent studies underscore the applicability of this technique for sponges [270].

## 12. Summary

Sesquiterpenequinones represent a substance class with increasing pharmacological interest. The initial concentration of an interesting compound may be too low to be effectively tested in some biological and pharmacological assays. Thus, the total synthesis of terpenequinones has become attractive in order to obtain the required amounts of compounds natural product analogues with optimized biological properties. Consequently, the development of these marine natural products is highly desirable and worthwhile from the viewpoint of medicinal chemistry and pharmaceuticals. Therefore, total synthesis of natural products will surely continue to be central to confirmation of natural product structure assignment, as well as providing material for biological testing towards pharmaceutical development, and investigations of biosynthetic pathways. 

The main routes to synthesize terpenequinones/hydroquinones include Diels-Alder cycloaddition reaction, coupling of the aldehydes with lithiated hydroquinone ether, radical decarboxylation and quinone addition reaction, Grignard reagent conjugated addition to α,β-unsaturated carbonyl group, reductive alkylation, cross-coupling reaction, furylation of quinones and furan polyene cationic cyclization. In addition, the application of cell culture for the production of bioactive compound from sponge is a promising way to utilize the bioactive potential of marine terpenoquinones sources.

Advances in total synthesis, especially function-oriented synthesis, biosynthetic technologies, primmorph models and genomic research offer new strategies for the medicinal chemical optimization of biologically active terpenequinones/hydroquinones. 
